# A Mathematical Model of the Metabolic and Perfusion Effects on Cortical Spreading Depression

**DOI:** 10.1371/journal.pone.0070469

**Published:** 2013-08-14

**Authors:** Joshua C. Chang, Kevin C. Brennan, Dongdong He, Huaxiong Huang, Robert M. Miura, Phillip L. Wilson, Jonathan J. Wylie

**Affiliations:** 1 Mathematical Biosciences Institute, The Ohio State University, Columbus, Ohio, United States of America; 2 Department of Neurology, University of Utah, Salt Lake City, Utah, United States of America; 3 Department of Mathematics, City University of Hong Kong, Kowloon Tong, Hong Kong; 4 Department of Mathematics and Statistics, York University, Toronto, Ontario, Canada; 5 Department of Mathematical Sciences and Center for Applied Mathematics and Statistics, New Jersey Institute of Technology, Newark, New Jersey, United States of America; 6 Department of Mathematics and Statistics, University of Canterbury, Christchurch, New Zealand; 7 Department of Mathematics, City University of Hong Kong, Kowloon Tong, Hong Kong; St. Joseph's Hospital and Medical Center, United States of America

## Abstract

Cortical spreading depression (CSD) is a slow-moving ionic and metabolic disturbance that propagates in cortical brain tissue. In addition to massive cellular depolarizations, CSD also involves significant changes in perfusion and metabolism—aspects of CSD that had not been modeled and are important to traumatic brain injury, subarachnoid hemorrhage, stroke, and migraine. In this study, we develop a mathematical model for CSD where we focus on modeling the features essential to understanding the implications of neurovascular coupling during CSD. In our model, the sodium-potassium–ATPase, mainly responsible for ionic homeostasis and active during CSD, operates at a rate that is dependent on the supply of oxygen. The supply of oxygen is determined by modeling blood flow through a lumped vascular tree with an effective local vessel radius that is controlled by the extracellular potassium concentration. We show that during CSD, the metabolic demands of the cortex exceed the physiological limits placed on oxygen delivery, regardless of vascular constriction or dilation. However, vasoconstriction and vasodilation play important roles in the propagation of CSD and its recovery. Our model replicates the qualitative and quantitative behavior of CSD—vasoconstriction, oxygen depletion, extracellular potassium elevation, prolonged depolarization—found in experimental studies. We predict faster, longer duration CSD *in vivo* than *in vitro* due to the contribution of the vasculature. Our results also help explain some of the variability of CSD between species and even within the same animal. These results have clinical and translational implications, as they allow for more precise *in vitro*, *in vivo*, and *in silico* exploration of a phenomenon broadly relevant to neurological disease.

## Introduction

Cortical spreading depression (CSD) is a self-propagated depolarization that occurs in the gray matter of many species [1]. In humans, it is known to occur during brain injury, stroke, and subarachnoid hemorrhage [2]. There is also strong evidence that CSD is responsible for migraine aura [3]–[5], a sensory hallucination associated with migraine attack.

Although CSD was discovered in 1944 by Leão [6], we still do not have a detailed understanding of how CSD is manifest. In particular, CSD has been associated with massive changes in cortical perfusion. The magnitudes of these changes vary by animal species, but significant decreases and increases in blood flow volume occur in all species tested [7]–[9]. Also common to all species tested is a mismatch in the delivery of substrates to meet metabolic demands, resulting in a derangement of neurovascular coupling [10], [11].

Blood delivery is known to play a significant role in CSD in several ways. Changes in perfusion can induce peri-infarct depolarizations (PID), which are electrophysiologically identical to CSD. Conditions that mimic the effects of hypoperfusion, such as oxygen glucose deprivation (OGD) and exposure to ouabain (an inhibitor of the 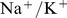
–ATPase), also generate spreading depolarizations [12]–[14].

Perfusion changes can also modulate the signature characteristics of CSD. Depending on the levels of the underlying oxygenation or blood pressure, the amplitude and duration of depolarization and the velocity of propagation of CSD can be altered [14], [15]. Clearly, CSD in vivo cannot be understood without reference to the vascular changes that condition – and are conditioned by – the phenomenon. There is a need to further explore the implications of the effects of perfusion and metabolism on various aspects of CSD.

Such explorations naturally lead to the development of mathematical models in which many mechanisms can be studied independently and/or simultaneously. Previous mathematical models of CSD have accounted for ionic diffusion [16], cellular membrane ionic currents [16], [17], the 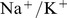
–ATPase and other membrane pumps [16], [17], and extra- and intracellular volume changes [18]–[20].

However, mathematical models of CSD have not looked at the dynamical implications of neurovascular coupling and metabolism. Here we formulate a five-compartment continuum model for CSD that uses known physiological data relating effective blood vessel diameter and extracellular potassium concentration to model oxygen delivery in the brain. The compartments in our model are: a somatic neuronal compartment, a dendritic neuronal compartment, an extracellular space compartment, a vascular tree compartment, and a glial compartment.

We show that the oxygen deprivation that results from both metabolic demand and vasoconstriction modifies the characteristics of CSD waves. Our results predict faster, longer duration CSD *in vivo* than *in vitro*, due to the contribution of the vasculature. Our results also help explain some of the variability of CSD between species and even within the same animal. In addition, the model explains differences between CSD *in vivo* and CSD in brain slices due to variant arterial constriction and dilation during the CSD event.

## Methods

**Figure 1:**
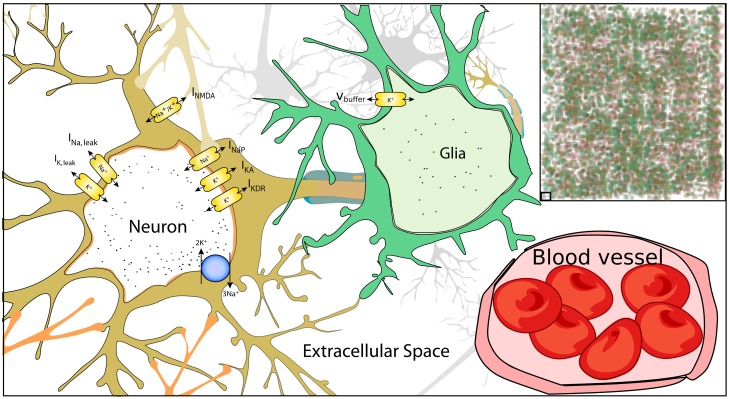
Diagram of the model. Neurons (mustard) consist of various sodium and potassium channels as well as the Na^+^ = K^+^–ATPase. Dendrites additionally consist of NMDA channels. Glia (teal) are incorporated as potassium buffers. Blood vessels (pink) bring in oxygen to supply the Na^+^/K^+^–ATPase. The cellular-level model is taken into the continuum limit (upper right) to yield a model with 5 compartments: neural cell bodies, neural dendrites, glial, vascular, and extracellular space.

To study the important new elements that affect and are affected by CSD, we formulate a five-compartment continuum mathematical model, see Fig. 1. Neurons comprise two of the five compartments: a compartment representing the dendritic processes (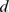), and a compartment representing the cell bodies (somatic compartment 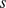). The ECS (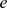), the vascular bed (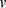), and glia (g) comprise the remaining compartments. Though cell swelling has been shown to occur during CSD [21], [22], we make the simplifying assumptions that the ICS and ECS volume fractions remain fixed, based on the findings of Yao et al. [19] and Bennett et al. [23] who found that osmotic effects do not significantly affect the propagation of CSD waves.

The ICS and ECS compartments include only the most relevant ions (sodium, potassium, chloride) and channels that have been shown to be responsible for the instigation and spread of CSD [17], [19], [24]. In the somatic membranes, we include P-type sodium channels, delayed-rectifier potassium channels, A-type potassium channels, and the 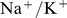
–ATPase. In the dendritic membranes, we additionally include NMDA channels, which for our model are permeable to sodium and potassium ions.

Our model assumes that the vascular compartment does not exchange fluid with the extracellular space. The effective diameters of proximal arterioles control the blood flow rate. In turn, the vascular diameters are coupled to neuronal activity through ECS potassium concentrations proximal to dendritic processes, which is also buffered by astrocytes.

### Membrane potential and ion transport using a neuronal model

The membrane potentials of the neuronal compartments, 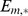 (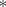 is either 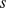 for somatic or 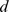 for dendritic), are governed by the coupled partial differential equations
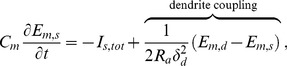
(1)

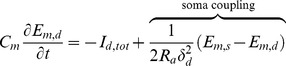
(2)where 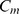 is the membrane capacitance per unit surface area (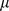 farad/cm^2^) for both the somatic and dendritic membranes, 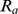 is the input resistance of the effective dendritic tree (ohms), 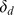 is the half length of the effective dendritic tree (cm), and 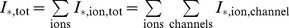 are the spatially-dependent total cross-membrane ionic currents per unit surface area (mA/cm^2^) for each neuronal compartment. Following Kager et al. [17], [20], [24] and Yao et al. [19], the total cross-membrane currents, 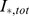, are given for the three major ions (sodium, potassium, and chloride) and are the sum of the active and passive (leak) sodium and potassium currents, the chloride (leak) current, and the sodium-potassium exchange pump current (see File S1).

The local rates of change of the ECS ions (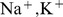, and Cl^−^) are due to membrane ionic currents, diffusion of extracellular ions, and the buffering of ECS potassium by glial cells. Note that all of the model differential equations have only time derivatives in them with the exception of the ECS diffusion equations for the ions. However, all of these equations depend implicitly on the spatial coordinate as a result of the spatial distribution of the ions.
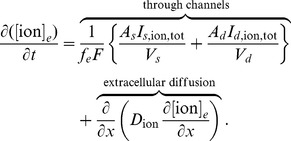
(3)The notation, 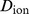, corresponds to the ion diffusion coefficient in aqueous solution taking into account tortuosity and volume fraction, see [25], [26], and 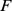 is the Faraday constant. The quantities 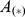 are the surface areas of the neuronal compartments in the total fixed volume given by the sum of the fixed somatic volume 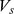, dendritic volume 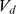, and extracellular volume, 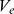. The ECS volume fraction is given by 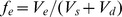. The equation for ECS potassium is modified by adding the buffering flux term, *v_buffer_*, given in the section *Potassium buffering by glial cells* later in this manuscript. The equations for the rates of change of ICS ions are
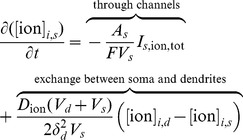
(4)

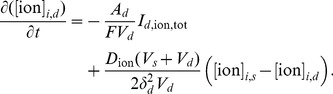
(5)Here we model the ion exchange between the somatic and dendritic portions of the neuron by a flux proportional to the difference between the ion concentrations. The exchange coefficient 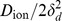 is estimated using the molecular diffusion coefficient and mean length of the dendrites, adjusted by the volume ratio of soma and dendrites. Note that we have not included the diffusion of ICS ions because any appreciable diffusion (relative to cell size) would require an ion to first become an ECS ion, diffuse extracellularly, and then enter into another cell to become an ICS ion.

As Cl^−^ is the only ECS anion in our simulations, to ensure electroneutrality, its initial extracellular concentration (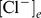) was determined by the sum of the concentrations of the major ECS cations: 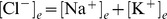. We chose the initial intracellular concentrations of chloride (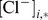) so that its Nernst potentials matched the resting membrane potential of 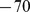 mV. To achieve intracellular electroneutrality in the soma and dendrites, we assume the existence of immobile anions. Although calcium can have major effects on neurotransmitter dynamics and other secondary messenger effects, we ignored the calcium concentration because of its relatively small values.

### Exchange pumps

During CSD, the ionic concentrations in the ECS and ICS are considerably displaced from steady-state. This displacement occurs primarily because of fluxes through voltage-gated sodium and potassium channels. Here, we include the sodium-potassium exchange pumps (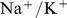
–ATPase) in the neuronal membranes, whose primary role is to restore the ionic concentrations back to their homeostatic state. The ionic pumps are active and consume energy. When local oxygen levels are depleted, ATP is in short supply. Therefore, the function of the ionic pumps in our model is related to oxygen consumption and vascular flows.

These pumps are involved in the movements of ICS sodium and ECS potassium against their electrochemical gradients and require active ionic pumps that consume energy. The pumps are fueled by the dephosphorylation of ATP in the cell [27] given by
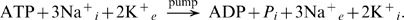
ATP is replenished by the reattachment of a phosphate ion to ADP and is powered by cellular respiration through both aerobic and anaerobic processes. When local oxygen in the tissue is very low, the normal ATP dynamics are perturbed.

The 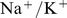
–ATPase is a transmembrane protein with two extracellular binding sites for potassium, three intracellular binding sites for sodium, and a single intracellular binding site for ATP. In each neuronal compartment, its potassium and sodium currents are given by 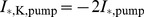 and 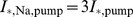 (as noted in the beginning of the previous section), 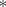 is either 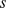 for somatic or 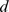 for dendritic), respectively, with
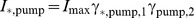
where
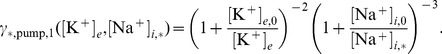
(6)
Eq. (6) is given in [17] and the expressions on the right are dependent on ECS potassium and ICS sodium concentrations. These equations implicitly assume that ATP is plentiful, which is not the case when oxygen is limited or when metabolic needs are high.

**Figure 2:**
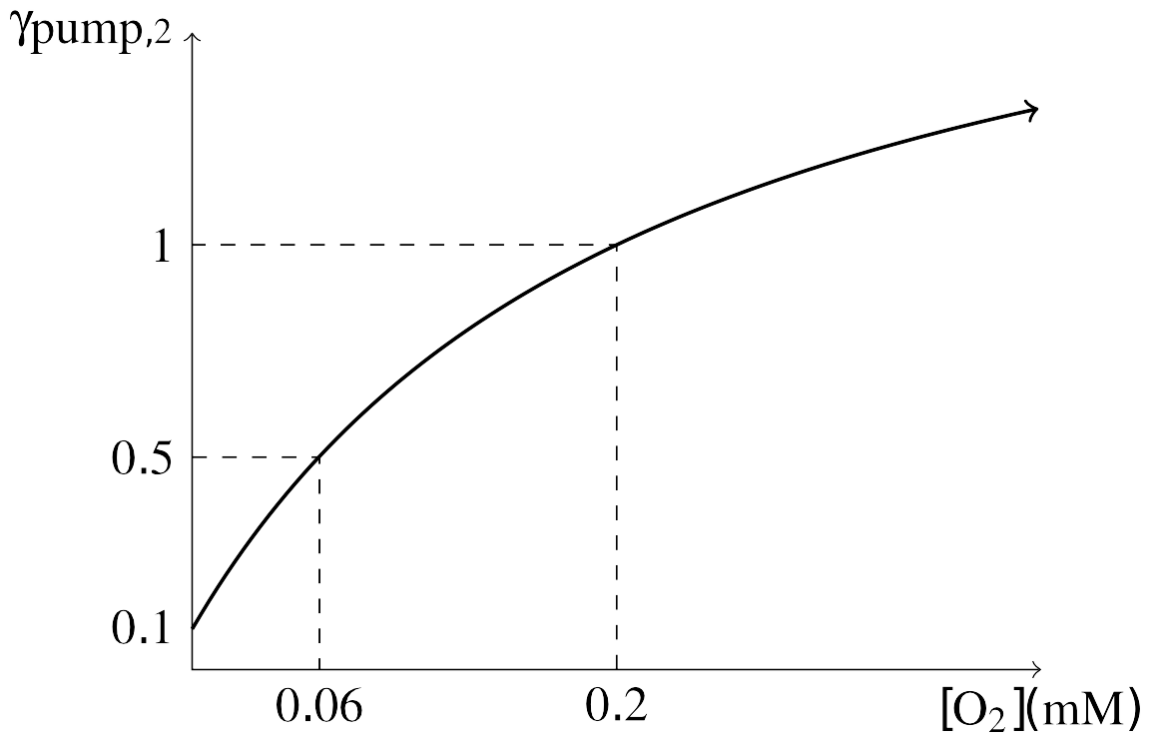
Oxygen availability affects the Na^+^/K^+^–ATPase. Shown is the relationship between tissue oxygen concentration and the oxygen-dependent portion of the pump rate (Eq (7)). Since some ATP is generated even in the absence of oxygen, the pump rate does not completely go to zero as the oxygen concentration approaches zero. The steady state oxygen concentration is 0.2 mM.

In the oxygen–limited regime, where ATP is limited, we modify this expression with an additional oxygen dependent term
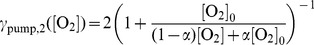
(7)where 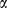 is the percentage (about 5%) of ATP production that is independent of oxygen [28], [29], the subscript 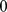 denotes the equilibrium values, and 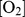 is the tissue oxygen concentration (see Fig. 2). This expression indicates that the pumping rate will be reduced whenever there is a decrease of the oxygen level in the tissue (see File S2 for details).

### Potassium buffering by glial cells

Astrocytes, a type of glial cell, play important roles in the instigation and propagation of CSD as well as in neurovascular coupling through neurotransmitter-mediated signaling pathways [30]. A principal role of astrocytes is the clearance of local increases of ECS potassium [31]. This buffering is achieved through a variety of inward rectifying potassium channels in the glial membrane and is bolstered by the extreme polarity of glial cell membranes with membrane potential near the Nernst potential for potassium [32]. For this study, we are not interested in the exact mechanisms of glial potassium buffering, but are interested in reproducing accurate potassium dynamics for our model. Thus, we incorporate astrocyte effects through empirical potassium buffering.

Following Kager et al. [17], we modeled the potassium-buffering flux, 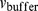, by the following differential equation,
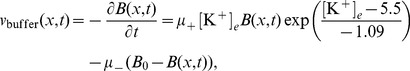
(8)where 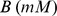 is the free buffer concentration, the rate constants 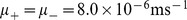 determine the speed at which potassium is buffered, and 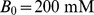 is the effective total buffer concentration. This equation describes strong buffering of extracellular potassium for concentrations above 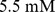, but is limited by saturation of the finite buffer. As the amount of buffered potassium increases, re-release of potassium into the extracellular space becomes more favorable. The initial value of the free buffer concentration is set to maintain steady state when the extracellular potassium concentration is at its rest value (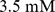).

### Neurovascular coupling and oxygen supply

We now describe how the tissue oxygen level is affected by and influences CSD. First, we assume that there exists an effective blood vessel radius 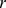 in the tissue, and that the cerebral blood flow rate (CBF) is given by
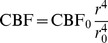
(9)in which the equilibrium values are again denoted by the subscript 0. This expression is based on the empirical observation that blood flow through the small vessels, where nutrient exchange primarily occurs, can be modeled as Poiseuille flow where the volume flow rate is proportional to 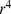 [33].

**Figure 3:**
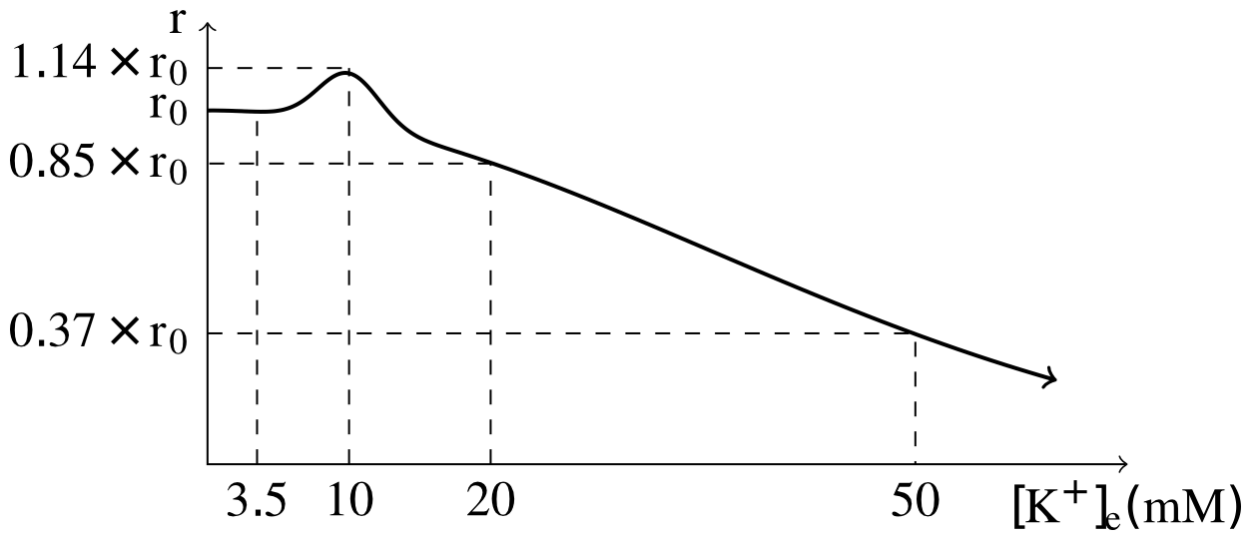
Relationship between vascular caliber and [K^+^]_*e*_. Effective vascular radius *r* as a function of ECS potassium concentration [K^+^]_*e*_.

We use an empirical model for the effective vessel radius 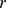, based on replicating the activity observed in many experimental studies on the subject [34], [35]. Extracellular potassium is known to dilate vessels at lower concentration elevations (less than 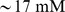), and constrict vessels at higher concentration levels. To mimic this vascular response, Farr and David [36] constructed a plot of the radius of cerebral arterioles versus extracellular potassium based upon currents through potassium channels in the membranes of vascular cells. To reproduce this plot, we assume that the effective vessel radius is given by
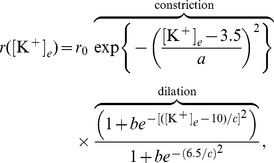
(10)which is a product of constricting and dilating terms. The parameter 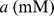 controls the constriction response, 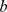 controls the amount of maximal dilation in the vessel, and 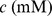 controls the dilation response. We found values of 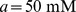, 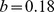, 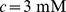 in Eq. (10) to mimic the plot of Farr and David, given by Fig. 3. We can use this expression in Eq. 9 to give a simple relationship for the CBF in terms of the extracellular potassium concentration.

We model the temporal evolution of the tissue oxygen concentration 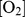, using a reaction-diffusion equation
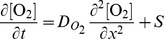
(11)with nonlinear source term given by
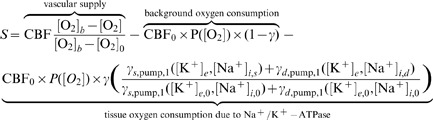
(12)where
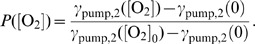
The first term on the right hand side of Eq. (12) is the amount of oxygen transferred from the blood stream to the tissue and is given by the product of CBF and the normalized concentration difference in oxygen tension between the blood and the tissue. The second term on the right represents the consumption of oxygen by the sodium-potassium exchange pump and other cellular processes that are assumed to remain steady during CSD. The pump consumption is given by the product of the equilibrium CBF and pump rate normalized by the steady-state pump rate. A fraction 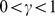 of the total oxygen consumption at steady-state is due to the pump. Experimental estimates of this parameter have ranged from as low as 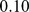 [37] to as high as 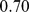 [38]. The final term in the source term is the consumption of tissue oxygen beyond their steady state value by 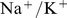
–ATPase. We include simulations over the full spectrum for thoroughness. Note that we have 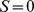 at steady-state.

## Results

We performed one-dimensional simulations by breaking up our model into a system of ODEs solved using Matlab routine ode15s with reflecting boundary conditions. The computational domain was set to a length of 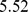 cm and discretized into 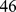 grid points. This domain was sufficiently long for the propagating wave to become stable and for boundary effects to remain insignificant. We used a minimal amount of potassium to induce CSD, finding that for injections of Gaussian boluses with 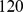 micron width, a 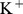 concentration of 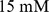 was sufficient to induce CSD. This concentration is near the previously-reported ceiling for potassium concentration in a non-CSD brain [39], [40] and confirmed to us that our phenomenological potassium buffer was behaving in a physiologically realistic manner.

Throughout these results, we report the duration of the CSD event. We measured the duration of potassium elevation by taking the total amount of time that 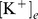 is above 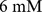. The transient sodium channel had negligible effect on the ionic currents during CSD (data not shown), so we chose to omit it from these simulations.

### Oxygen-clamped simulations

**Figure 4:**
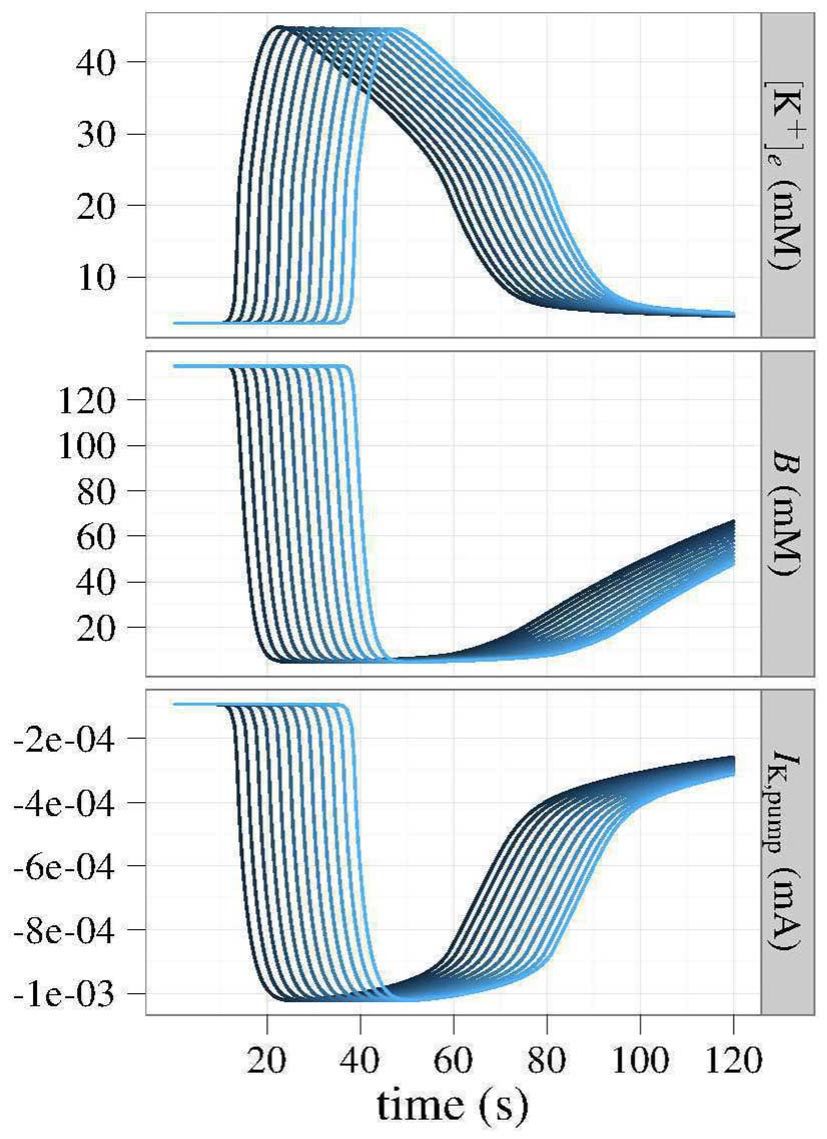
CSD in absence of oxygen consumption. Shown are the time-courses of the propagating waves of ECS potassium concentration, free buffer capacity, membrane potential, and Na^+^/K^+^–ATPase pump rate at positions 120 microns apart for simulations performed in the absence of oxygen consumption by the Na^+^/K^+^– ATPase. The Na^+^/K^+^–ATPase operates at rates determined solely by [K^+^]_*e*_ and [Na^+^]_*i*_.

First, we simulated our model with a fixed oxygen concentration, i.e., 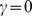 (see Eq (12)). Thus, the 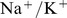
–ATPase does not consume any oxygen, and we assume that the tissue oxygenation is held at its steady state value. In this situation, we obtain CSD waves with a velocity of 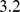 millimeters per minute and with ECS potassium concentration increasing to a maximum value of 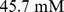. This result is similar to ECS elevations reported elsewhere in the literature [41]. The total duration of the elevated ECS potassium concentration is 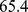 seconds (Fig. 4).

The potassium buffer acts similar in that in Kager et al. [17]. It saturates rapidly, after which it is responsible for a net re-release of potassium into the extracellular environment. The buffer acts this way in all simulations as it is independent of the oxygen level in our model. For this reason, we omit further mention of the potassium buffer.

### Blood vessel-clamped simulations

Next, we simulated our theoretical CSD where the effective blood vessel diameter remained fixed, thereby fixing the maximal oxygen flux rate into the tissue. We varied the oxygen coupling parameter 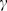 between 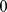 (no oxygen consumption by pump) and 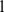 (maximal oxygen coupling) in increments of 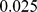.

**Figure 5:**
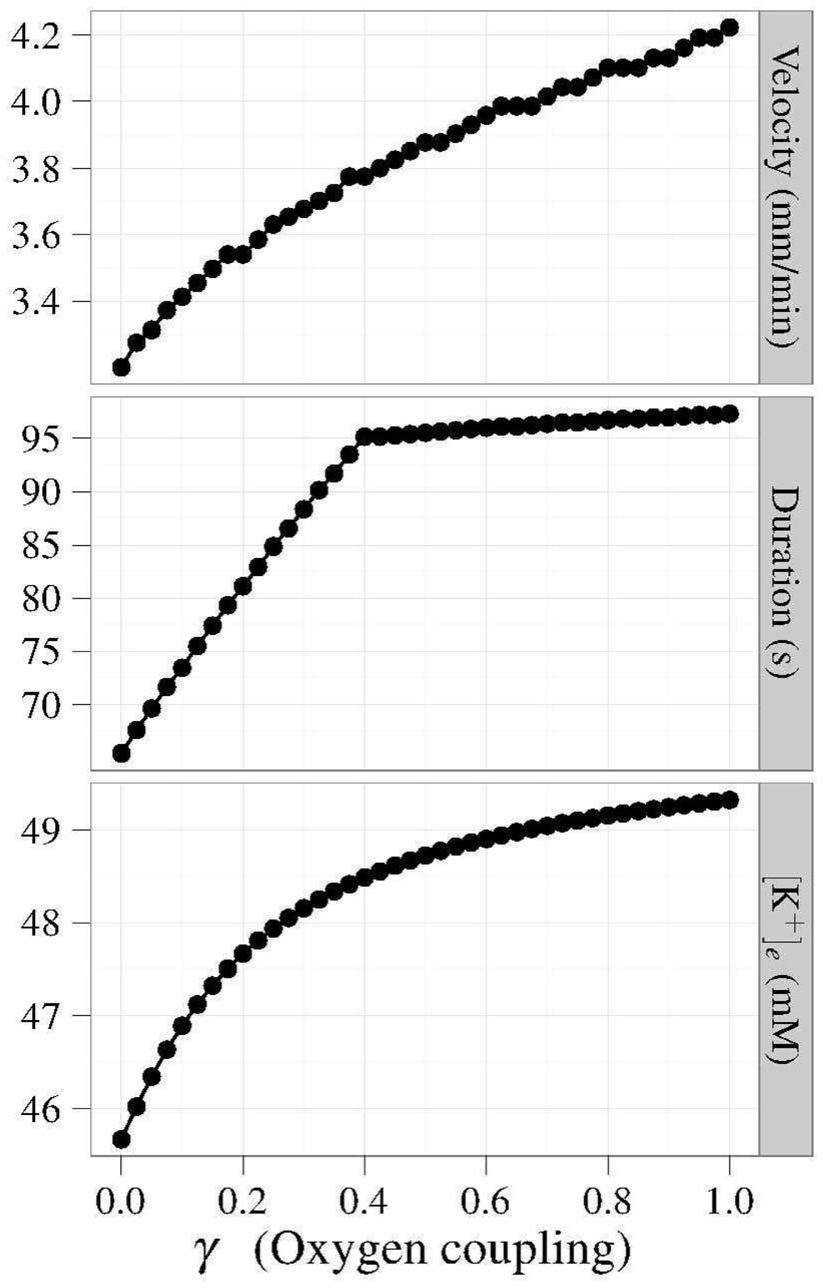
Oxygen coupled to Na^+^/K^+^–ATPase. Fixing the effective vascular radius while coupling oxygen to the Na^+^/K^+^–ATPase gives a purely consumption-based view of oxygen during CSD. Defining coupling as the fraction of oxygen consumed by the pump at steady-state, these simulations show that all of the CSD characteristics – speed, duration, maximum [K^+^] – increase with an increase in the coupling constant. The duration of the wave increases linearly from γ = 0 to γ≈0.4 before increasing linearly at a different rate when γ>0.4.

**Figure 6:**
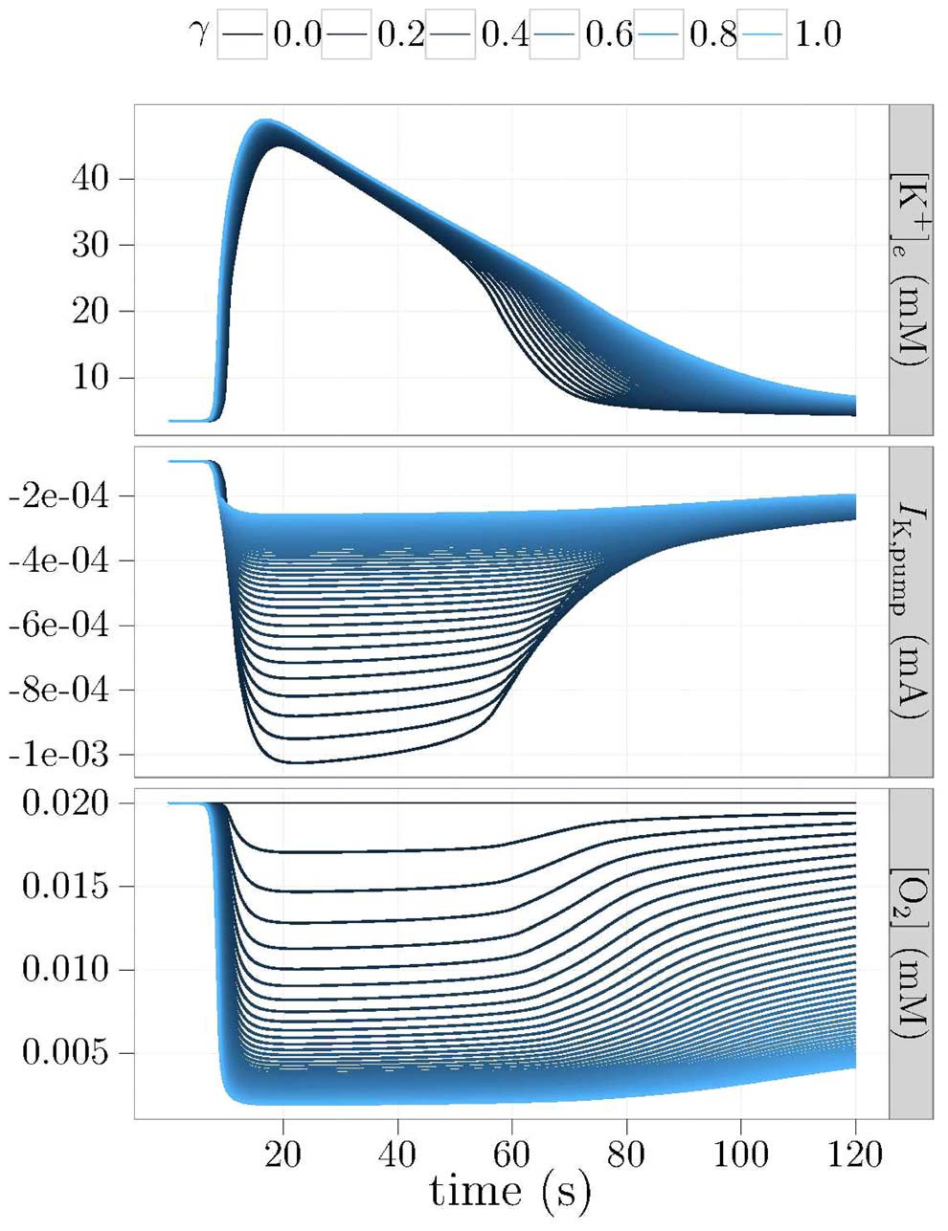
Fixed vascular caliber. Time courses at a fixed position, 780 µm downstream of the original stimulus. These simulations show the effects of oxygen consumption on CSD for a range of the oxygen coupling parameters, γ. Increasing γ prolongs the duration and magnifies the amplitude of the CSD because it implies that the pump consumes more of the available oxygen, thereby resulting in larger oxygen depletions.

The results shown in Fig. 5 illustrate the response of CSD to oxygen coupling. By increasing 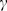, we increase the velocity, duration, and amplitude of the CSD waveform. To get a deeper understanding of these results, Fig. 6 shows the macroscopic observables, with curves parameterized by 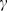. One sees that increases in 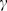 lead to greater drops in oxygenation. This deoxygenation results in decreases in the magnitude of the sodium-potassium–ATPase pump current, resulting in longer tails of recovery for both membrane potential and extracellular potassium concentration.

### Coupled vasculature

From the previous results, one sees that oxygenation has a definite impact on susceptibility of the tissue to CSD, as defined by its wave propagation speed, and on its recovery. The next question is whether vasoconstriction has an impact on CSD. We performed a series of simulations where we activated the vascular coupling that we defined in Eqs. (10) and (12). We simulated an idealized vascular response, using parameters mimicking the vascular behavior of Farr and David [36]. In these simulations, we varied the oxygen coupling parameter, 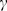, in order to see the variability in behavior one might expect to see in an idealized CSD experiment.

**Figure 7:**
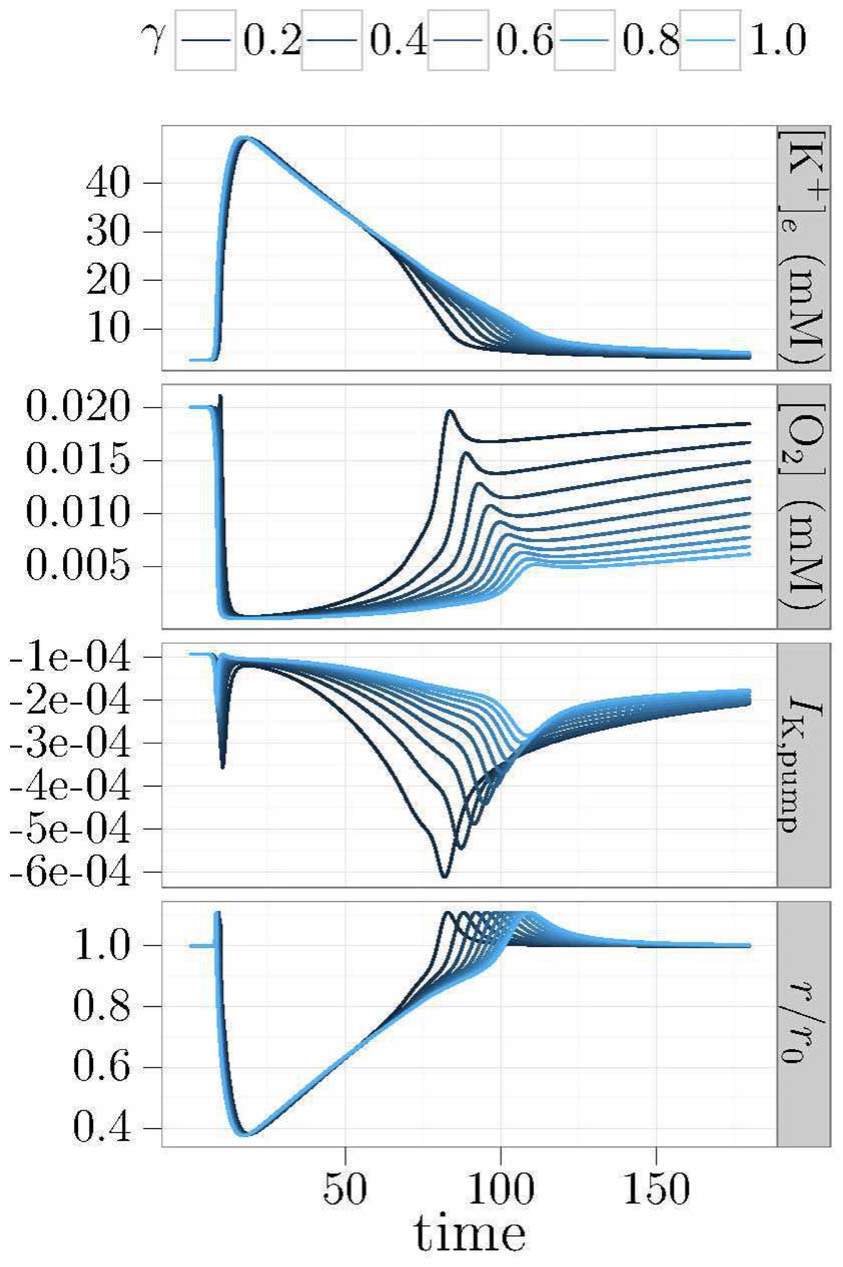
“Typical” vascular coupling. Time courses at a fixed position, 780 µm downstream of the original stimulus. Simulations performed using vessel of Farr and David [36]. After a short dilation period, the vessels constrict significantly to about 40% of their rest radii, before recovering.

The results, shown in Fig. 7, illustrate the effects of vascular coupling. One can see that the effective blood vessel radius drops rapidly to approximately 40% of its original value. This drop leads to steep sustained reductions in the oxygen concentration during the metabolic challenge from the CSD wave. The deoxygenation is reflected in the pump rate, where a large increase in inward potassium current at the beginning of CSD is rapidly diminished as oxygen is depleted. The implications of this chain of events are visible in the ECS potassium profile.

**Figure 8:**
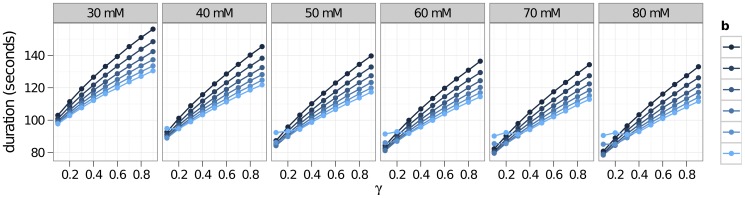
Duration of CSD for different vascular responses. Shown are CSD durations plotted against percent dilation *b* (the maximum vasodilation), constriction parameter *a* (this parameter from Eq. (10) has the units of mM and is the width of a Gaussian curve that controls how fast *r* drops as [K^+^] increases), and oxygen coupling constant γ. Duration is defined by the length of time that potassium concentration is elevated to a level greater than 6 mM. Increasing *a*, (shown from 30–80 mM) decreases the amount of constriction, resulting in quicker recovery from CSD. Likewise, increasing *b*, which increases the maximum dilation of the vessels, also reduces the duration of CSD.

**Figure 9:**
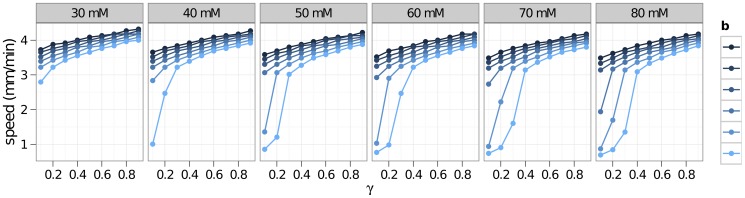
Speed of CSD for different vascular responses. Increasing the constriction parameter *a* makes the vessel less sensitive to potassium, weakening the resulting constriction. The speed decreases as *a* increases from 30 mM up to 80 mM, but increasing the amount of dilation by increasing *b* seems to have a greater effect.

As we noted earlier, the vascular response is variable and depends on factors such as species, individuals, and metabolic states. In Fig. 8, the effects of this variability are seen in simulations across a grid of values for the parameters 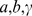 in Eq. (10). The duration of the CSD event decreases as the parameter 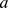 (ECS 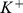 concentration at 63% constriction) and the parameter 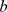 (percent maximal dilation) are increased. In Fig. 9, we have plotted the speed of CSD waves across a spectrum of possible vascular responses. These vascular responses are parameterized mainly by the two parameters, 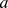 and 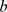.

## Discussion

Since its discovery, CSD has been known to involve massive changes in vascular caliber, and hence, perfusion [6]. Up to now, these vascular changes, which can have profound effects on cortical function and thus on CSD itself, have not been incorporated into CSD models. Using our model, we were able to examine the effects of vascular activity on CSD.

### Relationship between vascular activity and CSD

Our numerical experiments confirmed that oxygen delivery plays a significant role in the dynamics of CSD. Even in the absence of constriction, oxygen depletion is seen. Additionally, vasoconstriction and vasodilation were seen to further modify the characteristics of the CSD wave, particularly in the low-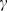 regime. By varying the relationship between vasoconstriction and ECS potassium, we achieved a continuum of CSD responses that could explain real physiological variability across species, within members of the same species, and for the same animal.

#### CSD uncoupled from vascular activity

Our model of CSD with fixed oxygen concentration resembles current CSD models, which rely on similar assumptions and conductances. Such models are relevant to CSD in *ex vivo* brain slices, where the perfusing medium has contact with an essentially unlimited source of oxygen. In this case, the pump is always able to perform at a rate determined by 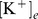 and 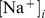, and the total pump current is constrained only by the total number of available pumps present, provided that there is sufficient nutrient available. Our model predicts that CSD waves propagate more slowly and recover more quickly in slices than *in vivo*.

#### CSD coupled with vascular activity

Coupling an inert vasculature with CSD, one obtains a sense of the metabolic demands that CSD places on the cortex. The large displacements of sodium and potassium require the use of energy to return the cortex to homeostasis. The physical constraints placed on blood delivery by the physiologically reasonable assumption of finite volume-flow-rate were seen to result in depletion of oxygen and reduction in potassium flux through the pumps. This finding is consistent with *in vivo* measurements of tissue oxygen and mitochondrial redox state – even with mild dilation. Several studies have found that both variables moved into a more-reduced oxidative state [21], [42]. In our model, the depletion of oxygen is due to increased metabolic demand driven by the 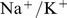
–ATPase.

#### The effects of vasoconstriction/vasodilation

Furthermore, we show that vasoconstriction can both decrease the tissue's ability to slow down the CSD wave, and impair its ability to recover. These effects are due to the reductions in blood flow causing a significant additional drop in cortical oxygenation levels. In Fig. 7, the vessel is seen to constrict to approximately 40% of its original radius. This constriction is within the experimental range reported in Chang et al. [10]. Due to the power law relationship between blood flow and effective blood vessel radius, even a small reduction in effective radius has large blood flow implications. A 60% constriction results in blood flow dropping to 2.6% of its original value. This effect is visible in the oxygenation level, which undergoes further decreases. Our simulations show that both wave speed and recovery time increase when vascular caliber is reduced.

Our simulations also show that vasodilation plays a role in CSD. Predictably, vasodilation appears to precondition the tissue so that it is better able to withstand the increase of ECS 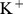 that accompanies the CSD wave. Due to the power law relationship, an increase of 14% in the vessel radius results in a 70% increase in blood volume flow rate. The result shown in Fig. 9 is a tissue that is better able to withstand potassium elevations, thereby causing slow-downs in the CSD wave. Vasodilation (beyond the effective resting radius) seems to play a significant role in the recovery from CSD, causing decreases in the recovery time (Fig. 8).

### Clinical and translational implications

If a major goal of CSD modeling is to understand its role in human disease, incorporation of perfusion and metabolism is an essential step. CSD is a near-complete leveling of ionic gradients which challenges the ability of homeostatic mechanisms to compensate. CSD is also triggered by changes in perfusion and metabolism. Finally, during CSD, the relationships between brain activity, perfusion, and metabolism - neurovascular coupling - are altered. These complex relationships can be expected to lead to a variety of responses, depending on the state of the brain tissue surrounding the depolarization. Biological data from animals and humans bear out this complexity and variability. Spreading depolarizations can be relatively innocuous - repetitive CSD in mouse over several days appears to cause no overt injury [43]. However, they can also be quite harmful, enlarging infarct and contusion areas in both animals and humans [14], [44]. These deleterious effects are almost certainly due to alterations in the vascular response to tissue depolarization, and thus cannot be understood from a modeling standpoint without explicit incorporation of perfusion and metabolism.

Our model more realistically represents conditions observed in the brain during experimental CSD and the spreading depolarizations of migraine and brain injury. Though it simplifies a complex vascular/metabolic response, it has the distinct advantage of making specific, quantifiable predictions which can be used to generate hypotheses for further experimentation. A particular advantage is the ability to explore the whole “CSD/metabolic parameter space,” which is not possible in biological experiments. This modeling could have important implications for study of the role of CSD in migraine, as the conditions which could generate such a massive depolarization in an awake behaving person remain obscure.

### Assumptions, limitations, and future directions

In this study, in order to make our model as widely applicable as possible, we have not considered the geometry of any particular vascular network. Our lumped model is assumed to be a good approximation of oxygen delivery dynamics in the brain, in general, for gray-matter. Further insight into the system may be gained by targeting specific tissue types for simulation.

Our simplified model relating arterial effective diameter to 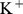 is likely applicable during CSD but does not mechanistically explain all the subtleties of neurovascular coupling under more normal conditions. Though 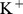 is involved in the coupling of neural and astrocytic activity to blood flow [45], other mediators including arachidonic acid derivatives, purines, nitric oxide, and possibly neurotransmitters are involved as well [7], [30], [46]. For the purposes of CSD, however, the supra-physiological swings in ECS potassium likely provide a good leading-order approximation of vascular behavior. Many of these other mediators also work via their effects on potassium channels [36].

Swelling of individual cells [17], [18], [21], [22], [47] and the tissue as a whole [10] appears to occur during CSD. Whether cell-swelling plays an important role in CSD is a controversial topic, as recent computational studies have cast some doubt on its impact [19], [23]. For this reason, we have omitted cell swelling from our model.

Finally, this model is not equipped to account for the complexity of the post-CSD state. Chang et al. [10] found that extracellular 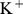 levels remained constant during a long–lasting hypoperfusion and depolarization that was found to follow CSD. As this phase may be clinically relevant, future modeling will focus on trying to understand the 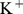
–independent mechanisms involved in the etiology of this period.

## Supporting Information

File S1 Cross-membrane currents and parameter values
(PDF)

File S2 Derivation of oxygen-dependent model for the Na^+^/K^+^ATPase
(PDF)

Table S1 Parameter values for active membrane ionic currents, from [1], [4]. Units are given in Table S2.
(PDF)

Table S2 Initial resting values and other relevant parameter values for the computations, following Kager et al. [1], [4].
(PDF)
