# Supplementary material for: A Mathematical Model of the Metabolic and Perfusion Effects on Cortical Spreading Depression
Source: PLoS One. 2013 Aug 14;8(8):e70469. doi: 10.1371/journal.pone.0070469 (PMC3743836; doi:10.1371/journal.pone.0070469)
Supplement: File S1 — Cross-membrane currents and parameter values (PDF) [file pone.0070469.s001.pdf]

Material S1 - Cross-membrane currents and parameter values

We assume that the membrane currents in the soma used by Kager et al. [1] apply here. The total cross-membrane current in the soma is given by the sum of the active and passive sodium, potassium, chloride, and nonspecific ionic currents. The sodium current is $I_{s,\mathrm{Na,tot}} = I_{s,\mathrm{Na,P}} + I_{s,\mathrm{Na,Leak}} + I_{s,\mathrm{Na,Pump}}$ where $I_{s,\mathrm{Na,P}}$ is the persistent sodium current, $I_{s,\mathrm{Na,leak}}$ is the sodium leak current, and $I_{s,\mathrm{Na,pump}}$ is the sodium current through the pump. Note that we have removed $I_{s,\mathrm{Na,T}}$, the fast transient sodium current, since this current was shown in [2] to not make any fundamental difference in the way CSD propagates. The active and passive potassium currents are $I_{s,\mathrm{K,tot}} = I_{s,\mathrm{K,DR}} + I_{s,\mathrm{K,A}} + I_{s,\mathrm{K,leak}} + I_{s,\mathrm{K,pump}}$ where $I_{s,\mathrm{K,DR}}$ is the potassium delayed rectifier current, $I_{s,\mathrm{K,A}}$ is the transient potassium current, $I_{s,\mathrm{K,leak}}$ is the potassium leak current, and $I_{s,\mathrm{K,pump}}$ is the potassium current through the pump. The passive chloride leak current is $I_{s,\mathrm{leak}}$. The mathematical expressions for these channels do not differ across the dendritic and somatic compartments so we omit the compartment prefix (s, d) from this point forward. In the dendritic compartment, the NMDA currents, $I_{d,\mathrm{Na,NMDA}}$ and $I_{d,\mathrm{K,NMDA}}$, are added to the total current.

The cross-membrane currents are modeled using the Goldman-Hodgkin-Katz (GHK) formulas for the active membrane currents given by

$$I_{\mathrm{ion,GHK}} = m^p h^q \frac{g_{\mathrm{ion,GHK}} F E_m \left[[\mathrm{ion}]_i - \exp\left(-\frac{E_m}{\phi}\right) [\mathrm{ion}]_e \right]}{\phi \left[1 - \exp\left(-\frac{E_m}{\phi}\right) \right]}, \tag{1}$$

where the permeability is absorbed into the parameter $g_{\mathrm{ion,GHK}}$. The factors in the parameter $\phi = RT/F$ are $R=$ 8.31 mV coulomb/mmol K, the universal gas constant, $T = 310$ K, the absolute temperature, and $F=$ 96.485 coulomb/mmol, the Faraday constant. The GHK equation is suitable when there is a large difference in concentrations between the ICS and ECS compartments, as argued by Koch and Segev [3]. For the passive leak currents, we used the Hodgkin-Huxley (HH) model given by

$$I_{\mathrm{ion},HH} \quad = \quad g_{\mathrm{ion},HH} \left(E_m - E_{\mathrm{ion}} \right). \tag{2}$$

In these general expressions for the GHK and HH types of currents, $g_{\mathrm{ion,GHK}}$ and $g_{\mathrm{ion,HH}}$ are the conductances associated with the channels for ion = Na^+ and K^+, and m and h are the activation and inactivation gating variables, respectively, for the different GHK-modeled channels that are ion-specific. Note that the $g_{\mathrm{ion,HH}}$ conductances for the sodium and potassium leak currents and the g_{leak} conductance for the chloride leak current are assumed to be constant. The variables, E_{ion}, are the Nernst potentials for ion = Na^+ and K^+ given by

$$E_{\mathrm{ion}} = \phi \log \frac{[\mathrm{ion}]_e}{[\mathrm{ion}]_i}. \tag{3}$$

For the chloride leak current, the equivalent Nernst potential is -70mV.

The gating variables, m and h, satisfy the following relaxation equations

$$\frac{dm}{dt} \quad = \quad \alpha_m(1 - m) - \beta_m m, \tag{4}$$

$$\frac{dh}{dt} \quad = \quad \alpha_h(1 - h) - \beta_h h, \tag{5}$$

and the values of α and β are given in Table S1, along with the exponents p and q [4]. The extracellular volume is assumed to be 15% of the intracellular volume, i.e., $V_e = 0.15V_i$.

We use the following procedure to choose the initial (equilibrium) values for the gating variables and ion concentrations. We first set the membrane potential at $E_m = -70$ mV and the sodium and potassium concentrations (listed in Table S2), from which we compute the parameters α and β (using the formulas in Table S2). We then compute the equilibrium values of m and h as

$$m = \frac{\alpha_m}{\alpha_m + \beta_m}, \quad h = \frac{\alpha_h}{\alpha_h + \beta_h}.$$

Next we choose the leak conductances, $g_{\text{Na,leak}}$ and $g_{\text{K,leak}}$, by setting

$$I_{\text{Na,tot}} = I_{\text{K,tot}} = 0.$$

Finally, we determine the chloride leak conductance in I_{leak} by assuming that $g_{\text{leak}} = 10g_{\text{Na,leak}}$.

The initial resting ion concentrations consistent with those in [1,4] are obtained by modifying the Na^+/K^+ exchange pump function and running the model equations until a steady state is reached. These values are given in Table S1 together with other parameter values.

References

1. Kager H, Wadman W, Somjen G (2000) Simulated seizures and spreading depression in a neuron model incorporating interstitial space and ion concentrations. Journal of Neurophysiology 84: 495-512.

2. Yao W, Huang H, Miura RM (2010) A continuum neuronal model for the instigation and propagation of cortical spreading depression. Bulletin of Mathematical Biology 73: 2773-2790.

3. Keener J, Sneyd J (2009) Mathematical Physiology: I: Cellular Physiology. Springer Verlag.

4. Kager H, Wadman W, Somjen G (2002) Conditions for the triggering of spreading depression studied with computer simulations. Journal of Neurophysiology 88: 2700-2712.
