# Supplementary material for: A Mathematical Model of the Metabolic and Perfusion Effects on Cortical Spreading Depression
Source: PLoS One. 2013 Aug 14;8(8):e70469. doi: 10.1371/journal.pone.0070469 (PMC3743836; doi:10.1371/journal.pone.0070469)
Supplement: File S2 — Derivation of oxygen-dependent model for the Na+/K+ATPase (PDF) [file pone.0070469.s002.pdf]

Material S2 - Derivation of the Na^+/K^+–ATPase oxygen–dependent model

The Na^+/K^+–ATPase is a transmembrane protein with two extracellular binding sites for potassium, three internal binding sites for sodium, and a single intracellular binding site for ATP. Its activity is well described by a sixth-order Hill-like equation of the form

$$I_{\mathrm{pump}} = I_{\max}\left(1+\frac{[\mathrm{K}^+]_{e,0}}{[\mathrm{K}^+]_e}\right)^{-2}\left(1+\frac{[\mathrm{Na}^+]_{i,0}}{[\mathrm{Na}^+]_i}\right)^{-3}\left(1+\frac{[\mathrm{ATP}]_{i,0}}{[\mathrm{ATP}]}\right)^{-1}. \quad (1)$$

To use this formulation for the pump activity, one needs to understand ATP dynamics within the cell. Fortunately, the biochemical pathways through which ATP is generated are well known [1, 2]. However, these pathways are complicated, involving a panoply of intermediate products and enzyme-substrate interactions. The full details of the biochemical pathway are not relevant towards achieving an understanding of ATP availability during CSD. The authors in [1,2] provide an ATP mass-balance equation that is a sum of aerobic pathways, anaerobic pathways, and consumption by pumps. It takes the form

$$\frac{\partial[\mathrm{ATP}]}{\partial t} = \left(\underbrace{v_{\mathrm{mito}}}_{\mathrm{aerobic}} + \underbrace{v_{\mathrm{CK}} + v_{\mathrm{PGK}} - v_{\mathrm{HK}} - v_{\mathrm{PK}}}_{\mathrm{anaerobic\ processes}} - v_{\mathrm{pump}}\right)\left(1-\frac{d\mathrm{AMP}}{d\mathrm{ATP}}\right)^{-1}, \quad (2)$$

where v_{mito} is oxygen dependent and takes the form

$$v_{\mathrm{mito}} \propto \left(1+\frac{[\mathrm{O}_2]_0}{[\mathrm{O}_2]}\right)^{-1}.$$

These studies focus on ATP:AMP ratios in the regime where the reaction catalyzed by the adenylate kinase enzyme is fast relative to other reactions. In neurons, we do not expect the adenylate kinase reactions to dominate due to the presence of aerobic processes.

Instead, we looked at the system of equations presented in these studies to find the behavior when the system is dominated by aerobic production of ATP and consumption of ATP. In this regime, the inter-conversion between ADP and ATP dominates over the conversion of AMP to ADP, so that $d\mathrm{AMP}/d\mathrm{ATP} \ll 1$. We also note that the oxygen-dependent ATP-production by the mitochondria (v_{mito}) is a fast reaction, thereby yielding the quasi-steady-state relationship between ATP and O_2, $\mathrm{ATP} = \mathrm{const}_1 + \mathrm{const}_2\left(\frac{\mathrm{O}_2}{\mathrm{const}_3+\mathrm{O}_2}\right)$, where const_1 refers to anaerobic ATP production.

References

1. Cloutier M, Bolger F, Lowry J, Wellstead P (2009) An integrative dynamic model of brain energy metabolism using in vivo neurochemical measurements. Journal of Computational Neuroscience 27: 391–414.

2. Heinrich R, Schuster S (1996) The Regulation of Cellular Systems. Springer.

3. Kager H, Wadman W, Somjen G (2000) Simulated seizures and spreading depression in a neuron model incorporating interstitial space and ion concentrations. Journal of Neurophysiology 84: 495-512.

4. Kager H, Wadman W, Somjen G (2002) Conditions for the triggering of spreading depression studied with computer simulations. Journal of Neurophysiology 88: 2700-2712.
