# Supplementary material for: A Mathematical Model of the Metabolic and Perfusion Effects on Cortical Spreading Depression
Source: PLoS One. 2013 Aug 14;8(8):e70469. doi: 10.1371/journal.pone.0070469 (PMC3743836; doi:10.1371/journal.pone.0070469)
Supplement: Table S1 — Parameter values for active membrane ionic currents, from [1], [4]. Units are given in Table S2. (PDF) [file pone.0070469.s003.pdf]

Table S1

Table S1. Parameter values for active membrane ionic currents, from [1,2]. Units are given in Table S2.

Currents mA/cm^2	$g_{ion,GHK}$ mA cm	Gates $m^p h^q$	Voltage-Dependent Rate Constants
$I_{\mathrm{Na,P}}$	2 $\times 10^{-6}$	m^2h	$\alpha_m = \frac{1}{6(1+\exp[-(0.143E_m+5.67)])}$<br>$\beta_m = \frac{\exp[-(0.143E_m+5.67)]}{6(1+\exp[-(0.143E_m+5.67)])}$<br>$\alpha_h = 5.12 \times 10^{-8}\exp[-(0.056E_m + 2.94)]$<br>$\beta_h = \frac{1.6\times10^{-6}}{1+\exp[-(0.2E_m+8]}$
$I_{\mathrm{K,DR}}$	10 $\times 10^{-5}$	m^2	$\alpha_m = 0.016\frac{E_m+34.9}{1-\exp[-(0.2E_m+6.98)]}$<br>$\beta_m = 0.25\exp[-(0.25E_m + 1.25)]$
$I_{\mathrm{K,A}}$	1 $\times 10^{-5}$	m^2h	$\alpha_m = 0.02\frac{E_m+56.9}{1-\exp[-(0.1E_m+5.69)]}$<br>$\beta_m = 0.0175\frac{E_m+29.9}{\exp(0.1E_m+2.99)-1}$<br>$\alpha_h = 0.016\exp[-(0.056E_m + 4.61)]$<br>$\beta_h = \frac{0.5}{1+\exp[-(0.2E_m+11.98)]}$
I_{NMDA}	1 $\times 10^{-5}$	mh	$\alpha_m = \frac{0.5}{1+\exp\left(\frac{13.5-[\mathrm{K}^+]_e}{1.42}\right)}$<br>$\beta_m = 0.5 - \alpha_m$<br>$\alpha_h = \frac{1}{2000\left(1+\exp\left[\frac{[\mathrm{K}^+]_e-6.75}{0.71}\right]\right)}$<br>$\beta_h = 5 \times 10^{-5} - \alpha_h$

References

1. Kager H, Wadman W, Somjen G (2000) Simulated seizures and spreading depression in a neuron model incorporating interstitial space and ion concentrations. Journal of Neurophysiology 84: 495-512.

2. Kager H, Wadman W, Somjen G (2002) Conditions for the triggering of spreading depression studied with computer simulations. Journal of Neurophysiology 88: 2700-2712.
