# Supplementary material for: A Mathematical Model of the Metabolic and Perfusion Effects on Cortical Spreading Depression
Source: PLoS One. 2013 Aug 14;8(8):e70469. doi: 10.1371/journal.pone.0070469 (PMC3743836; doi:10.1371/journal.pone.0070469)
Supplement: Table S2 — Initial resting values and other relevant parameter values for the computations, following Kager et al. [1], [4]. (PDF) [file pone.0070469.s004.pdf]

Table S2

Table S2. Initial resting values and other relevant parameter values for the computations, following Kager et al. [1,2].

Parameter	Value	Unit
R_a (input resistance of dendritic tree)	1.83×10^5	ohms
d_s (diameter of soma)	5.45×10^{-4}	cm
d_d (diameter of dendrite)	9.39×10^{-5}	cm
δ_d (half-length of dendrite)	4.5×10^{-2}	cm
A_s (surface area of soma)	1.586×10^{-5}	cm^2
A_d (surface area of dendrite)	2.6732×10^{-4}	cm^2
V_s (volume of soma)	2.160×10^{-9}	cm^3
V_d (volume of dendrite)	5.614×10^{-9}	cm^3
C_m(membrane capacitance)	7.5×10^{-5}	s /Ω cm^2
I_{max} (Na^+/K^+–ATPase rate)	1.48×10^{-3}	mA / cm^2
E_m	-70	mV
$[K^+]_e$	3.5	mM
$[K^+]_i$	133.5	mM
$[Na^+]_e$	140	mM
$[Na^+]_i$	10	mM
$[O_2]_0$	2×10^{-2}	mM
CBF_0	2.5×10^{-2}	mM/s
D_{O_2}	5×10^{-4}	cm^2/s
$[O_2]_b$	4×10^{-2}	mM

References

1. Kager H, Wadman W, Somjen G (2000) Simulated seizures and spreading depression in a neuron model incorporating interstitial space and ion concentrations. Journal of Neurophysiology 84: 495-512.

2. Kager H, Wadman W, Somjen G (2002) Conditions for the triggering of spreading depression studied with computer simulations. Journal of Neurophysiology 88: 2700-2712.
